# Probiotic-Induced Tolerogenic Dendritic Cells: A Novel Therapy for Inflammatory Bowel Disease?

**DOI:** 10.3390/ijms22158274

**Published:** 2021-07-31

**Authors:** Shaghayegh Baradaran Ghavami, Hamid Asadzadeh Aghdaei, Dario Sorrentino, Shabnam Shahrokh, Maryam Farmani, Fatemeh Ashrafian, Maria Pina Dore, Shahrbanoo Keshavarz Azizi Raftar, Seyed Mobin Khoramjoo, Mohammad Reza Zali

**Affiliations:** 1 Basic and Molecular Epidemiology of Gastrointestinal Disorders Research Center, Research Institute for Gastroenterology and Liver Diseases, Shahid Beheshti University of Medical Sciences, Tehran 19839-63113, Iran; Sh.bghavami@yahoo.com (S.B.G.); hamid.assadzadeh@gmail.com (H.A.A.); shabnamshahrokh@gmail.com (S.S.); maryam_farmani4@yahoo.com (M.F.); mobin.khoramjoo@gmail.com (S.M.K.); 2 IBD Center, Division of Gastroenterology, Virginia Tech Carilion School of Medicine, 3 Riverside Circle, Roanoke, VA 24016, USA; 3 Department of Clinical and Experimental Medical Sciences, University of Udine School of Medicine, 33100 Udine, Italy; 4 Microbiology Research Center, Pasteur Institute of Iran, Tehran 13169-43551, Iran; fatemeh.ashrafian24@gmail.com (F.A.); hediehk90@gmail.com (S.K.A.R.); 5 Department di Medical, Surgical and Experimental Sciences, University of Sassari School of Medicine, 07100 Sassari, Italy; mpdore@uniss.it; 6 Gastroenterology and Liver Diseases Research Center, Research Institute for Gastroenterology and Liver Diseases, Shahid Beheshti University of Medical Sciences, Tehran 19839-63113, Iran; nnzali@hotmail.com

**Keywords:** inflammatory bowel diseases, ulcerative colitis, Crohn’s disease, probiotics, dendritic cells, mucosal tolerance

## Abstract

Inflammatory bowel diseases (IBDs) are immune-mediated, chronic relapsing diseases with a rising prevalence worldwide in both adult and pediatric populations. Treatment options for immune-mediated diseases, including IBDs, are traditional steroids, immunomodulators, and biologics, none of which are capable of inducing long-lasting remission in all patients. Dendritic cells (DCs) play a fundamental role in inducing tolerance and regulating T cells and their tolerogenic functions. Hence, modulation of intestinal mucosal immunity by DCs could provide a novel, additional tool for the treatment of IBD. Recent evidence indicates that probiotic bacteria might impact immunomodulation both in vitro and in vivo by regulating DCs’ maturation and producing tolerogenic DCs (tolDCs) which, in turn, might dampen inflammation. In this review, we will discuss this evidence and the mechanisms of action of probiotics and their metabolites in inducing tolDCs in IBDs and some conditions associated with them.

## 1. Introduction

Inflammatory bowel diseases (IBDs)—ulcerative colitis (UC) and Crohn’s disease (CD)—are increasing worldwide [1,2]. The causes of IBDs are still unknown, but—among others—gut microbiota have been shown to play a critical role in the immune alteration in IBD patients [3]. Therapies for IBD have evolved from relatively simple anti-inflammatory medications (aminosalicylates and corticosteroids) to immunomodulators to specific molecules targeting selected pro-inflammatory secretion pathways, e.g., tumor necrosis factor (TNF-α), interleukins (IL) IL-12/IL-23, and Janus kinases [4,5]. In addition, a number of different novel molecules targeting a variety of mechanisms are currently being tested/developed. Among them, some capitalize on our knowledge of the immunopathology of IBDs [6].

Dendritic cells (DCs) are important for inducing both immunity and tolerance, and they are also known as strong inducers of regulatory T cells (T-reg) [7,8]. Their capacity to increase T-reg populations has been used for the treatment of a number of immune-mediated diseases including IBDs, rheumatoid arthritis (RA), multiple sclerosis (MS), and type 1 diabetes (T1D) [9].

Tolerogenic DCs (tolDCs) can be induced by antigen-dependent signals leading cells to express semi-mature costimulatory molecules (CD80, CD86) and stimulate the production of anti-inflammatory cytokines [7]. It has been demonstrated that tolDCs can be generated by several immunosuppressive agents including some probiotics [10]. However, their tolerogenic function must be focused on a causative antigen. At present, the antigen(s) involved in IBD is(are) still unknown. Hence, finding suitable antigens to which tolerance can be induced is one of the greatest challenges in DC therapy. In this review, we will discuss how probiotics might react with pattern-recognition receptors (PRRs) on immature DC, thereby inducing tolDCs and which target might be the most effective to impact the mucosal immune homeostasis.

## 2. Immunopathogenesis of IBD: Main Players

**Figure 1:**
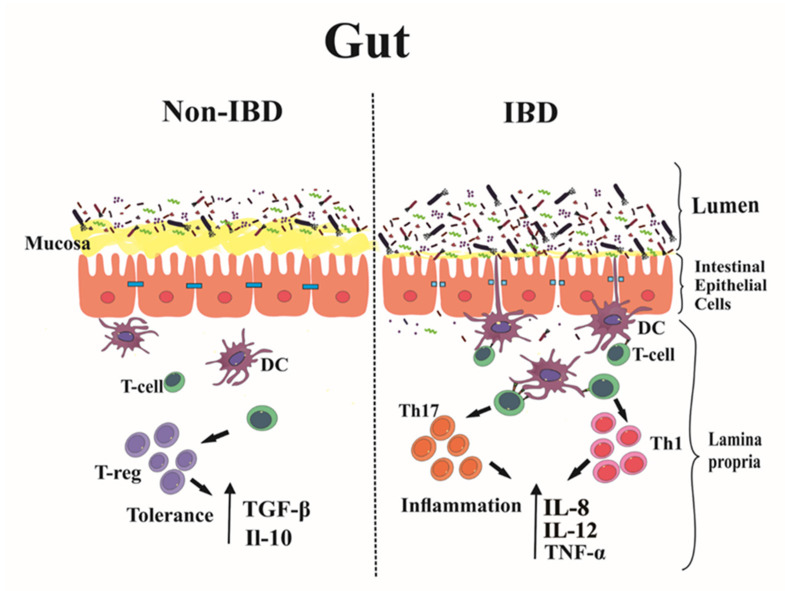
Early events in inflammatory bowel disease (IBD). During the early stages of IBD, an imbalance between the immune system response and the physiological gut inflammation results in breakage of the mucosal barrier. This leads to increased intestinal permeability, antigen translocation to the lamina propria and into the circulatory system, and an increased number of resident immune cells (dendritic cells, macrophages, Th1, Th2, Th17, and B cells) which encounter the intestinal bacteria, leading to progression of inflammation.

The line between tolerance and inflammation of the gastro-intestinal immune system is very narrow. When immune tolerance is disturbed, inflammation might ensue, as for example, in IBDs [11]. IBDs are chronic intestinal inflammatory diseases characterized by different profiles of inflammatory molecules and proinflammatory cytokines [12]. In these diseases, initial damage to the mucosal barrier and the intestinal epithelium leads to an increased intestinal permeability. This, in turn, facilitates the exposure of intestinal bacteria, pathogens, and food antigens to immune cells, such as neutrophils, macrophages, and DCs [13], a process that causes intestinal inflammation [14]. The critical role of the innate immune system is to regulate the cellular PRRs’ expression to preserve tolerance against commensal bacteria thus preventing inappropriate immune responses. By contrast, the adaptive immune system consists of T and B cells [15] that play a well-known role in the progression of chronic inflammation in IBD (Figure 1) [11].

A robust body of research has shown that an imbalance between Th1 and Th2 subsets might play a crucial role in the etiology and pathogenesis of a number of diseases including IBDs. While UC mostly displays a Th2 response (with its derived cytokines IL-4, IL-5, and IL-13), CD is characterized by an atypical Th1 response involving cytokines (IL-12, IFN-ɣ, TNF-α, and IL-1) different from UC [16]. Programmed death-ligand 1 (PD-L1)—also known as cluster of differentiation 274 (CD274) or as B7 homolog 1 (B7-H1)—is a protein encoded by the CD274 gene and promotes the secretion of proinflammatory cytokines, including TNF-α and IFN-γ, by DCs of IBD patients, which play an essential role in the pathogenesis of these diseases. Moreover, PD-L1 is also involved in the progression of CD [17].

Intestinal epithelial cells (IECs) are involved in digestion and absorption of food-derived products and protect the organism from microbial infection. In addition, IECs produce high levels of the IL-1, a family of cytokines that result in downregulation of the retinoic acid signaling pathway and in the activation of the inflammation pathways [18,19].

Toll-like receptors (TLRs) are a class of proteins that play a significant role in the innate immune system. They are membrane-spanning receptors usually expressed on macrophages and DCs and are able to recognize structurally conserved molecules derived from microbes. Several studies conducted in IBD patients have shown that an overexpression of TLRs, such as TLR2 and TLR4, and the upregulation of adhesion molecules in the endothelium, might contribute to and promote inflammation by inducing the production of pro-inflammatory cytokines such as TNF-α, IL-1β, and IL-8 [14,20].

Lymphocyte Th17 plays a critical role in suppressing the activity of T-reg cells [21]. For instance, in the mucosa and serum of CD and UC patients, a greater percentage of Th17 cells (and its signature cytokine IL-17A) has been detected compared with healthy subjects [21].

Colonic mucus and anti-microbial peptides are altered during disease activity in IBD, possibly due to the abnormalities of tight junction proteins in epithelial cells; this allows bacteria to reach the epithelium, affecting the absorption of food and the handling of microbial products [22].

It has been suggested that the resolution of inflammation and the repair process of the intestinal mucosa in IBD involves an increase in the effector T-cells-to-T-regs ratio [23,24].

DCs play a vital role in the tolerance process. In the so-called central tolerance, they control the elimination (by a negative selection) of self-reactive T cells in the thymus and by inducing T-regs. In the peripheral tolerance, tolDCs induce maintenance of immune homeostasis and break the self-tolerance of CD4+ T cells, which might, otherwise, result in autoimmunity [25]. Homing markers on DCs in IBD patients play an important role in the regulation of inflammation in the gut [26]. The DCs in the gut reduce the expression of the skin homing markers CLA and CCR4 in UC patients while inducing the expression of the colon homing marker CCR9 and β7 integrin in the gut [27,28]. In CD, mucosal DCs express more CD40 with an increased production of IL-6 and IL-12 [26]. In both CD and UC, mucosal DCs overexpress TLR2 and TLR4 [28]. By contrast, conventional DCs (cDCs) are a group of DCs lying in the gastro-intestinal tract. These cells are also defined as sCD103+ mucosal dendritic cells—which are heterogeneous populations subdivided into CD11b+ and CD11b− subsets [29]. The CD103+ CD11b+ subset is more abundant in the small intestine than in the colon. Such a subtype is decreased by over 75% in the inflamed and uninflamed intestinal tissue in CD patients compared to controls [17].

Plasmacytoid dendritic cells (PCDs) are another group of DCs—chemokine dependent—present in the inflamed gut, although they do not appear to play a critical role in IBD pathogenesis [30].

The activation of intestinal CD103+ of IBD patients results in the upregulation of microbial recognition receptors. Hence, local changes in the gut microbiota may alter the balance and regulation signals received by mucosal DCs. Finally, activated DCs—compared to resting DCs—are able to produce inflammatory cytokines [31]. In summary, it is clear that DCs play a fundamental role in CD and UC pathogenesis. Therefore, targeting their regulation could provide a tool to reduce gut inflammation.

## 3. Tolerogenic DC Therapy

**Figure 2:**
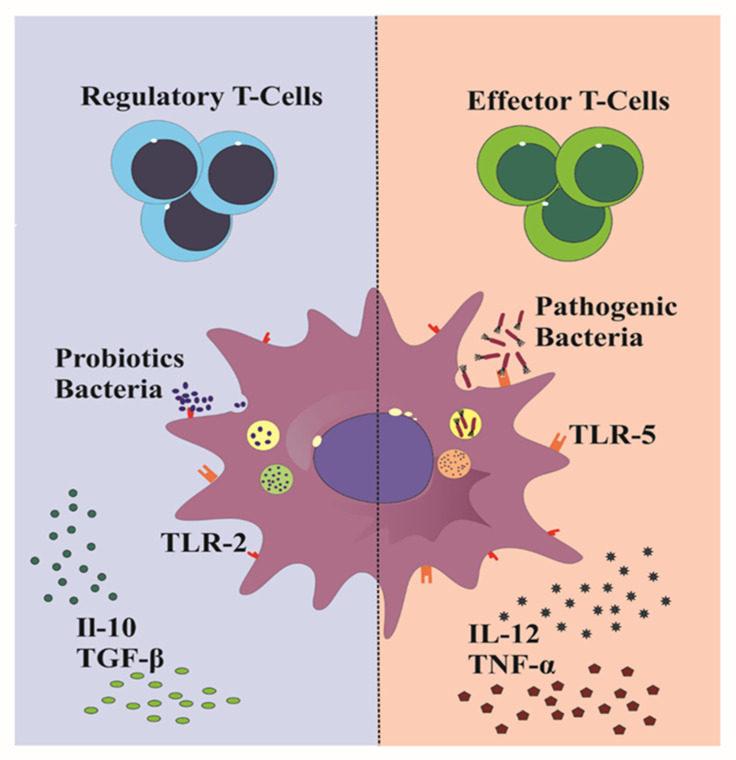
The maturation pathway of dendritic cells. The dendritic cell (middle) drives the development of effector T cells or T-regs. The response against the microbiota-derived antigens regulates the phenotypic expression of costimulatory (CD80/CD86) molecules through Toll-like receptor (TLR) ligands and specific cytokine release from dendritic cells. Tolerogenic dendritic cells might shift the balance to restore self-tolerance and reach long-term remission in autoimmune and immune-mediated diseases.

Tolerogenic DCs (tolDCs) have regulatory functions and play a fundamental role in immune tolerance. They are characterized by a semi-mature phenotype expressing costimulatory molecules (CD80/CD86) that can differentiate through TLR ligands or when exposed to a specific cytokine environment [32]. In addition, they express immunomodulatory molecules and produce immunosuppressive factors. The semi-mature, costimulatory CD80/CD86 signals strongly influence the proliferation and differentiation of T-regs acting through CD28 molecules on T cells, which, in turn, leads to the activation of anergy-associated genes inducing immune tolerance [33,34]. Among immunomodulatory molecules and anti-inflammatory cytokines expressed by tolDCs able to inhibit proinflammatory immune responses there are PD-L1, Ig-like inhibitory receptors IL-T3 and IL-T4, IDO, nitric oxide (NO), IL-10, and TGF-β [33]. It is crucial that DCs go through the immature state to act as tolDCs [35]. Several antigens are capable of inducing DC maturation that might trigger effector T cells or tolerance T cells (Figure 2).

Once they develop into tolDCs, these cells produce immunomodulatory factors that result in the expansion of T-regs. For this reason, tolDCs have been used to restore self-tolerance to achieve long-term remission in autoimmune and immune-mediated diseases [36]. The treatment of most autoimmune and immune mediated diseases involves anti-inflammatory therapies and systemic immunosuppressive, both often causing severe systemic side effects. Targeted or tolerance-inducing antigen-specific therapy (“transtolerance”) might represent an attractive alternative. In this regard, tolDCs have been shown to suppress the autoreactive T cell response and induce immune tolerance in autoimmune diseases. For effective tolDC immunotherapy, a specific antigen must be targeted to restore long-term antigen-specific tolerance, while avoiding generalized immunosuppression [37,38]. Most commonly, autologous tolDCs are generated from peripheral blood monocytes following ex vivo generation in GM-CSF and IL-4 cell culture medium. tolDC therapy has already been tested in a number of autoimmune and immune-mediated diseases including rheumatoid arthritis (RA), CD, multiple sclerosis (MS), and type 1 diabetes (DM1). Many factors influence tolDCs’ efficacy, including the route, dose, and duration of administration. However, the choice of an antigen relevant in the control of DC maturation is the single most important factor [39]. Such an antigen has been identified in autoimmune diseases associated with autoantigens—for example, the basic protein transgenic myelin oligodendrocyte glycoprotein (MOG) in MS. In DM1, the immune system recognizes glutamic acid decarboxylase 65 (GAD65) as a foreign antigen. A number of clinical trials in phase 1 and 2 of tolDC therapy have already been conducted in these conditions. Zubizarreta et al., in a clinical trial in phase I for MS, have shown that three doses of tolDCs administered by IV every two weeks were well tolerated and led to high production of IL-10 [40]. Other studies have shown that both intradermal and intranodal tolDCs administration are safe and well tolerated in MS and other conditions [41,42].

Other authors generated tolDCs by treating human MoDCs with NF-kB with four citrullinated peptide antigens, named “Rheumavax”. The treatment was well tolerated and led to an increase in T-regs a month after a single injection [43,44].

In the healthy status, the phenotypic DC subsets found in the intestinal mucosa maintain their tolerance, switching during infection or chronic IBD to a proinflammatory phenotype [45]. However, in IBDs, the specific autoantigen remains unidentified despite significant efforts. Hence, tolDC therapy in IBD has not significantly progressed compared to other immune-mediated diseases, and human studies are scarce. For example, Jauregui-Amezaga et al. have demonstrated that intraperitoneal administration of three doses of autologous tolDCs in CD patients was safe, although it did not impact on clinical outcomes [46]. A number of IBD animal models have been used to test tolDC therapy. The two most commonly used models are the dextran sulfate sodium (DSS) model for colitis and the 2,4,6-trinitrobenzene sulfonic acid (TNBS) model for CD [47]. In a TNBS-induced colitis model, the transfer of tolDCs treated with Vasoactive Intestinal Peptide (VIP) significantly improved the clinical and histopathology severity of colitis [48]. Additional studies have focused on pre-clinical models. For example, it has been shown that the tolerogenic phenotype of DCs is able to protect against TNBS-induced colitis in mice [46]. In another study, Engman et al. showed that injection of bone marrow-derived DC generated in the presence of a mixture of antisense DNA oligonucleotides targeting the primary transcripts of CD40, CD80, and CD86 prevented the progression of DSS-induced colitis. The mice also exhibited a significant increase in Foxp3+ T-regs and IL-10+ B-reg in MLN and spleen [49].

Additional studies have also shown that tolDCs secrete anti-inflammatory cytokines and modulate T-cells towards the development of Foxp3+ T-regs in the intestine of mice and humans [50,51]. This very preliminary evidence suggests that tolDC therapy could be very effective in IBD. However, finding suitable modulating antigens in IBD for DC differentiation remains a formidable challenge. Globally, these data indicate that the DC/T-reg/B regulatory axis plays a central role in the gut by (re)establishing tolerance and regulating T-regs.

## 4. Generation of tolDCs Ex Vivo

There are different ways to generate tolDCs.

### 4.1. Pharmacologically Modified tolDCs

DCs can be generated ex vivo from monocytes, and a number of strategies have been tested to induce tolDCs. These include modulation strategies by pharmacologic agents, such as dexamethasone, rapamycin, aspirin, cyclosporine, rosiglitazone, cocktails of immunomodulatory cytokines including IL-10, TGF-β, IL-6, and TNF-α; natural compounds [52,53,54]; short stimulation with microbial products, such as resveratrol, sulforaphanedihydroxy, vitamins, and lipopolysaccharide (LPS) [55,56]; by cell signaling inhibitors such as protein kinase C inhibitors or the CTLA4–Ig fusion protein abatacept [57]. In these induction models, the tolDCs’ efficacy has not been tested in vivo, particularly in inflammatory conditions such as IBD.

### 4.2. Probiotics as tolDCs Inducers

A number of probiotics have been used to produce tolDCs of clinical grade, potentially useful for the treatment of IBD [58,59]. Probiotics are live microorganisms that might be involved in the regulation, stimulation, and modulation of immune responses [60].

The immunomodulatory effect of probiotics occurs via interaction with enterocytes and DCs leading to regulation of the innate and adaptive immune systems [61]. Studies have shown that probiotic bacteria are capable of reacting with pattern recognition receptors (PRRs) on DCs that detect distinct evolutionarily conserved structures on pathogens, termed pathogen-associated molecular patterns (PAMPs), or by secreting soluble compounds, which consequently induce tolDCs [62,63]. Different genera, species, and strains of probiotics directly affect DCs’ maturation. Probiotics might regulate the levels of anti-inflammatory cytokines, such as IL-10 and TGF-β, and induce Tregs (Table 1).

PRR’s, such as TLRs, C-type lectin receptors (CLRs), retinoic acid-inducible gene 1 (RIG-I), or nucleotide-binding oligomerization domain (NOD), determine the DC maturation pathway towards the stimulation and polarization of naive T cells into T cell activation or T cell anergy, respectively [64,65].

Available evidence shows that DCs can be directly controlled by probiotic antigens. Two important strains of probiotics, which play a critical role in directing DCs’ differentiation into tolDCs, belong to the Lactobacillus spp. and to the Bifidobacterium spp. [58,66]. Studies have shown significant differences in the ability of different Bifidobacterium strains to activate tolerogenic DCs and induce naive differentiation of T cells [67]. Bifidobacterium infantis is a strain living near human epithelial cells, and it has immune-regulatory effects on epithelial cells, dendritic cells, and lymphocytes [63]. For example, B. infantis can modulate adaptive immune responses by inducing vitamin A and tryptophan metabolic pathways in DCs [68].

In a recent study, we showed that *B. bifidum* induces CD80 and CD86 expression in CD patients, and also increases IL-10 and TGF-β secretion in a dose-independent manner. By contrast, TLR expression was decreased by all probiotic bacteria with the exception of *B. bifidum* in DCs of UC patients [69]. Two additional species of Bifidobacterium have also been studied in this context: *B. longum* and *B. breve* C50. *B. breve* has been shown to possess various immunoregulatory effects on DCs and might play a key role in prolonging DCs’ survival through interaction with TLR2 and IL-10 secretion [70]. The *B. breve* demonstrated a marked tendency to reduce inflammation and to induce an increase of IL-10 production [58,71]. Moreover, the use of *B. breve* as probiotic supplementation in infants aged 0–27 days, was associated with a decreased risk of islet autoimmunity [72]. Overall, these studies provide evidence that these probiotics might play a crucial role in the maturation of DCs and could be used as target Ags in the treatment of inflammatory diseases. Other studies have shown that Bacteroides fragilis produces an immunomodulatory polysaccharide, called polysaccharide A (PSA), that plays a vital role in the prevention of TNBS-induced colitis in mice by inducing T-regs in a TLR2-dependent manner [73]. In addition, Shen et al. have shown that the PSA of *B. fragilis*-derived outer membrane vesicles (OMVs) was capable of preventing experimental colitis in mice by affecting DCs [74]. Yet, another study has shown that PSA prevents experimental colitis by interacting with ATG16L1 and NOD2 [75]. The OMVs of another Bacteroides species—Bacteroides thetaiotaomicron—have been shown to impact the balance of pro- and anti-inflammatory cytokines and ultimately ameliorate IBD [76]. Collectively, these studies show that OMVs released by probiotics (so-called post-biotics) can interact with DCs and reduce inflammation by affecting surface membrane molecules [77]. All these studies also demonstrate that delivered autologous monocyte-derived tolDCs treated with a specific antigen, such as influenza matrix peptides (MP), peptidoglycan, and glycoproteins with dexamethasone and vitD3, were safe and well tolerated [44]. In all cases, they increased the T-reg population in the patients’ peripheral blood [78,79].

In recent years, other approaches have been tested. Among them, the use of extracted cell wall components or non-viable whole microorganisms and fragments of bacterial cells (known as para-probiotics) has attracted attention as a potential inducer of tolerogenic immune responses for the treatment of inflammatory diseases [80,81,82]. For example, the treatment of *L. salivarius* Ls33 with peptidoglycan (PGN) appears capable of rescuing mice from colitis via the transfer of PGN-induced regulatory DCs [83]. *L. delbrueckii *is another species of Lactobacillus able to significantly decrease the expression of costimulatory molecules and surface markers in probiotic-induced mature DCs (MDCs), in both healthy subjects and in patients with systemic lupus erythematosus (SLE) [84]. In addition, Mazzeo et al. reported that *L. gasseri* could impact the DC maturation pathway not only by the production of tolDCs but also through changes in the protein secretome, reinforcing its anti-inflammatory role [85]. Two studies including UC patients and healthy controls showed that *L. plantarum* and *L. casei* were able to modulate DCs’ function and restoration and to control the intestinal immune response [86,87,88].

Components of Faecalibacterium prausnitzii interact with CD103+ DCs in the lamina propria, a process which, in turn, induces DC migration to mesenteric lymph nodes and the induction of T-regs. This bacterium also plays a role in Foxp3 + T-reg suppression by increasing IL-10 levels in APCs and subsequently inhibiting Th17 cells induced by inflammatory stimuli. Interestingly, the abundance of *F. prausnitzii* correlated negatively with the expression of TLR4 in intestinal DCs, an observation suggesting a close relationship of intestinal DCs with this bacterium, which can induce tolDCs and maintain immune homeostasis [89,90].

Probiotics are precursors of non-viable bacterial or metabolic products that also have bioactivity known as post-biotics. Post-biotics include secreted soluble compounds such as vitamins, SCFA, proteins, metabolites, and extracellular vesicles, among the others [91]. These compounds have immunomodulatory properties and, in some cases, they have been shown to be more active than the bacterium itself [45,92,93]. For example, soluble mediators derived from *L. rhamnosus *GG (LGG) appear capable of modulating DC function and inducing both Th1 and T cell regulatory phenotypes (Table 1) [62]. A recent study has shown that production of IL-10 is increased in monocytes/macrophages derived from DC/PBMC cultures treated with LGG and its soluble factors [93]. Mikulic et al. studied the impact of commensal *L. rhamnosus* on the responsiveness of DCs freshly recovered from mouse Peyer’s patches, mesenteric lymph nodes, and spleen. The probiotic led to an increased expression of TLR2, TLR4, and retinaldehyde dehydrogenase 2 on the mucosal DC, production of IL-10 and TGF-β, decreased surface expression of co-stimulatory markers, decreased IL-12 production, and induction of T-regs [94]. In addition, Mileti et al. have shown that *L. paracasei*, as a whole bacterium and as a supernatant of a centrifugate, can stimulate tolDCs and suppress inflammation [95]. In another study, it was shown that the surface (S) layer A protein (SlpA), a main antigen of *L. acidophilus*, attaches to DCs and induces the production of IL-10 while decreasing the production of IL-12p70 [96].

Overall, probiotic-secreted metabolites appear capable of affecting the function of DCs and could play a role in the treatment of a number of diseases [26,97].

OMVs (see above) are vesicles secreted by probiotics of nano-size and contain a wide range of components. They are essential to bacteria to communicate with each other or with the host [98]. A number of in vitro and in vivo studies have shown their anti-inflammatory and immunomodulatory properties [77,99]. For example, treatment with OMVs derived from Bacteroides fragilis induce tolDCs and T-regs and ameliorate colitis in mice [74]. In addition, membrane vesicles derived from Escherichia coli Nissle 1917 can modulate the function and maturation of DCs and induce a T-regs’ response in PBMC [100]. Overall, gut microbiota/probiotics-derived OMVs could potentially represent a new tolerogenic Ag for the treatment of inflammatory diseases, especially IBD. During the maturation process, DCs lose their capacity to acquire a soluble antigen, but they gain T cell stimulatory capacity to increase antigen processing and upregulation of MHC, costimulatory molecules, and cytokines [101]. Once maturation is achieved, tolDCs induced pharmacologically without an antigen might not be effective in controlling inflammation. As mentioned above, post-biotics also affect DCs’ function; for instance, STp derived from *L. plantarum* displays regulatory effects on colon DCs, stimulates T cells, and might play a role in controlling intestinal homeostasis in UC [26].

These substances might also change tolDCs into active DCs. Further studies are needed to determine which commensal/probiotic bacteria or non-viable forms of them are more effective in driving DCs’ differentiation into tolDCs and which ones are most effective in an inflammatory environment.

## 5. Other Conditions Related to IBD

### 5.1. Non-Alcoholic Fatty Liver Disease

The alteration of gut microbiota metabolism and the concomitant increased intestinal permeability in IBD might lead to hepatic infiltration of fat products and bacteria through the portal system [102]. This pathway is known as the gut–liver axis, and it is at the basis of the association of non-alcoholic fatty liver disease (NAFLD) and IBDs [103]. Lipids (especially cholesterol and triglycerides) accumulated in hepatic cells play a critical role in NAFLD [104]. As mentioned above, the alteration in gut microbiota might impact on host metabolism, nutrient absorption, and immune function. Thus, treatments able to manipulate the gut microbiota, such as probiotics or prebiotics, have been proposed to treat NAFLD [105]. For example, *Lactobacillus* and *Bifidobacterium* have been shown to be able to regulate lipid metabolism in NAFLD [106]. In addition, probiotics have been shown to decrease total cholesterol, low-density lipoprotein cholesterol, and triglycerides, all of which are main risk factors of NAFLD [107]. Hence, theoretical and limited practical evidence suggest that probiotics might be beneficial for hepatic cell protection. The mechanisms at the basis of these effects are multiple, the vast majority being related to the regulation of liver function, lipid profiles, plasma glucose profiles, and degree of liver fat infiltration.

### 5.2. Arachidonic Acid and the Cyclooxygenase-2 Pathway

Arachidonic acid (AA) is selective tumoricidal agent and has potential antimicrobial properties against a variety of bacteria. Cyclooxygenase-2 (COX2) metabolizes AA and produces prostaglandins (PGs) and thromboxane A2 [108]. Hence, AA metabolism is a double-edged sword, since it leads to both pro- and anti-inflammatory molecules. PGs can active the NFK-β pathway and increase pro-inflammatory cytokines such as IL-1 and TNF-α [109]. Because of this, overexpression of COX-2 can be associated with chronic inflammation, cancer, and suppression of apoptosis [110]. On the other hand, regulation of the COX2 pathway is very important for the conversion of AA to PGE2, which results in control of inflammatory pathways, mitogens, growth factors, and pro inflammatory cytokine secretion [111]. There is also evidence suggesting that COX-2 activity and PGE2 synthesis may be involved in the process of intestinal carcinogenesis. This seems to be particularly important since it is well known that IBD-related carcinogenesis occurs as a result of chronic inflammation [112]. Thus, a balanced expression of COX-2 is essential for intestinal homeostasis. Some probiotics—such as *Lactobacillus plantarum* and *Lactobacillus rhamnosus GG*—seem able to regulate COX2 expression leading to the inhibition of the inflammatory cascade and reduction of pro-inflammatory cytokines [113,114]. *Lactobacillus casei* has also been shown to downregulate COX-2 expression in a rodent trinitrobenzenesulphonic acid (TNBS) colitis model [115]. Interestingly, other studies have identified microbial factors, such as butyrate and propionate, that might promote intestinal homeostasis and downregulate COX-2 expression [116]. In general, this is a field that might provide an important contribution to our understanding of the mechanisms of intestinal inflammation and cancer and their prevention in IBD and other conditions.

## 6. Conclusions

An imbalanced immune response to dysbiosis seems to play an important role in the onset and progression of many inflammatory diseases, especially IBD. tolDC therapy or transtolerance could affect such a balance and re-establish immune homeostasis. Data show that ex vivo generation of tolDCs via exposure to a suitable antigen (intestinal self-antigen, probiotics, post-biotics, and para-probiotics) could represent a potential therapeutic tool to re-induce tolerance and ameliorate inflammation in a number of conditions.

## Figures and Tables

**Table 1:** Probiotics, post-biotics, and para-probiotics: tolDC induction and other effects.

Probiotics	Strain	Type of Treatment	Source of DC	Donor	Main Results	Reference
***L. gasseri***	OLL28099	Bacteria	Mouse BMDC	WT mice	Modulation of DCs’ maturation	Mazzeo et al. [85]
***L. delbrueckii***	subsp lactis	Bacteria	Human PBMC	Healthy volunteers and SLE patients	Induction of T-regs	Esmaili et al. [84]
***L. rhamnosus***	*GG*	Soluble mediators (LSM)	Human PBMC	Healthy volunteers	Modulation of DCs’ functions, induction of Foxp3+ T cells	Ludwig et al. [62]
		Soluble factors	Human PBMC	Healthy volunteers	Induction of IL-10 production in DCs; DCs’ immunomodulation	Fong et al. [93]
		Bacteria	Human PBMC	Healthy volunteers	Modulation of DCs’ function, induction of IL-10 production and T cell priming capability of DCs	You et al. [58]
***L. crispatus***	SJ-3C-US	Bacteria	Human PBMC	Healthy volunteers	Modulation of DCs’ function, induction of DCs’ maturation and high IL-10 production in DCs, induction of T-regs	Eslami et al. [87]
***L. plantarum***	*-*	STp	Human colonic DCs	UC patients	Modulation of DCs’ function and restoration of tolDCs in human gut DCs	Al-Hassi et al. [26]
	BMCM12	STp	Human PBMC	Healthy volunteers	Modulation of DCs’ phenotype and function, regulatory effects on gut DCs	Bernardo et al. [86]
***L. casei***	Shirota	Bacteria	Human PBMC	UC patients	Restoration of dysregulated DCs’ function in UC	Mann et al. [87]
***L. salivarius***	-	Bacteria	Human PBMC	CD and UC patients	Modulation of Crohn’s DCs, increased production of IL-10 and TGF-β, decreased production of IL-12	Ghavami et al. [69]
***L. paracasei***	B21060	Bacteria and its supernatant	Human PBMC	-	Stimulation of DCs and suppression of T cell inflammatory cytokine production	Mileti et al. [95]
***L. acidophilus***	NCFM	SlpA	Human PBMC	Healthy volunteers	Modulation of DCs’ and T cells’ functions, induction of IL-10 production in DCs	Konstantinov et al. [96]
***L. reuteri***	*-*	Bacteria	Human PBMC	Nd	Modulation of DCs’ function, DCs’ maturation, and induction of T-regs	Smits et al. [66]
***B. bifidum***	*-*	Bacteria	Human PBMC	CD and UC patients	Induction of CD80 and CD86 expression in CD patients. Induction of IL-10 and TGF-β production in a dose-independent manner	Ghavami et al. [69]
***B. longum***	*infantis* CCUG 52486	Bacteria	Human PBMC	Healthy volunteers	Modulation of DCs’ function, induction of IL-10 production and T cell priming capability of DCs	You et al. [58]
***B. breve***	C50v	Supernatant	Human PBMC	Healthy volunteers	Induction of maturation, activation, and survival of DCs survival through TLR2, and increased IL-10 production	Hoarau et al. [70,71]
***B. subtilis***	*-*	Bacteria	Human PBMC	CD and UC patients	Increased levels of TGF-β in DCs from UC patients	Ghavami et al. [69]
***B. coagulans***	*-*	Bacteria	Human PBMC	CD and UC patients	Increased levels of TGF-β in DCs from CD patients	Ghavami et al. [69]
***B. thetaiotaomicron***	VPI-5482	Freeze-killed bacteria	Human Colonic DC	Healthy volunteers	Increased levels of IL-10 compared to UC and CD patients	Durant et al. [76]
		PSA OMV	Human PBMC	Healthy volunteers UC and CD patients	Induction of IL-10 production in healthy colon and blood DCs and promotion of regulatory DC responses	
***B. fragilis***	NCTC9343	PSA OMV	Mouse BMDC	WT and colitis mice	Induction of tolDCs’ function, increased T-regs and anti-inflammatory cytokine production, and protection from colitis	Shen et al. [74]
***F. prausnitzii***	*-*	Bacteria	Human PBMC	Healthy volunteers	Modulation of DCs’ function, induction of tolDCs and T-regs, induction of IL-10 production	Alameddine et al. [89]
		Bacteria	Human PBMC	Healthy volunteers	Induction of IL-10 production and DCs’ maturation	Rossi et al. [90]
***E. coli Nissle***	1917	MV	Human PBMC	Healthy volunteers	Modulation of DCs’ function, DCs’ maturation and induction of T-regs response	Diaz-Garrido et al. [100]

SLE: Systemic lupus erythematosus, BMDCs: bone marrow DCs, MLNDCs: mesenteric lymph nodes DCs, WT: wild type mice, STp: serine–threonine peptide, PGN: peptidoglycan, Slp A: surface layer protein A, MV: membrane vesicles, OMV: outer membrane vesicles.
